# Measurement Invariance of the PROMIS Family Relationships Scale Among Autistic and General Population Adolescents

**DOI:** 10.1002/aur.70161

**Published:** 2026-01-08

**Authors:** Rachel M. Benecke, Zachary J. Williams, Laura Graham Holmes, Judith S. Miller, Elizabeth A. Kaplan-Kahn

**Affiliations:** 1 Center for Autism Research, Children’s Hospital of Philadelphia, Philadelphia, Pennsylvania, USA; 2 College of Education and Human Development, Temple University, Philadelphia, Pennsylvania, USA; 3 Vanderbilt University School of Medicine, Nashville, Tennessee, USA; 4 Silberman School of Social Work at Hunter College, City University of New York, New York, New York, USA; 5 Perelman School of Medicine, University of Pennsylvania, Philadelphia, Pennsylvania, USA

**Keywords:** adolescence, autism, family relationships, measurement invariance, patient-reported outcome measures, PROMIS

## Abstract

Social relationships are a key component of quality of life, a high-priority outcome for autistic people, and family relationships are critical in adolescence. The PROMIS Family Relationships scale has been well validated for use with the general population, but psychometric validation in the autistic population is lacking. This study investigated measurement invariance of the PROMIS Family Relationships among autistic and general population adolescents. The scale demonstrated scalar invariance between the groups, providing evidence that it measures the same construct equivalently and scores can be meaningfully compared between groups. With a well-validated self-report measure, researchers can ask autistic teens directly about their experiences of their family relationships, rather than relying solely on parent proxy report.

## 1 | Introduction

Autistic people and their families value quality of life (QoL) as a research priority and clinical outcome (Benevides et al. 2020; Frazier et al. 2018; Gotham et al. 2015; Pellicano et al. 2014). Among adolescents in general, one factor associated with more positive QoL is strong family relationships (Grevenstein et al. 2019). Members of the autism community agree that family relationships are also a key element of QoL for autistic people (Graham Holmes et al. 2020). Among autistic teens, family relationships are known to influence life satisfaction (Lam et al. 2020; Øverland et al. 2024) and transition-to-adulthood outcomes (Test et al. 2014). The importance of family relationships may be even more pronounced for autistic adolescents than their non-autistic peers (Krieger et al. 2018). However, there remain significant gaps in our field’s understanding of the role that family relationships may play in supporting the QoL of autistic adolescents.

A striking gap in the literature is a lack of studies utilizing self-report measures to evaluate autistic adolescents’ own perspectives on their family relationships. Research about autism and family relationships tends to focus on how parents or siblings relate to an autistic family member (e.g., Factor et al. 2019; Karst and Van Hecke 2012; Orsmond et al. 2009), rather than investigating this relationship from the autistic person’s perspective. When considering autistic teens’ perspective of family relationships, researchers often rely on parent-proxy reports (Schwartz et al. 2018), wherein the parent responds on behalf of their child. However, since adolescence is marked by individuation for autistic and non-autistic individuals alike (Test et al. 2014; Wood et al. 2018), parental insight into teens’ subjective experiences may be limited, and parent proxy report is inherently biased for the construct of family relationships. Therefore, to accurately understand teens’ perspectives of their family relationships, researchers should rely on self-report whenever possible.

Self-report is often overlooked in autism research (Schiltz et al. 2024). Although some have interpreted discrepancies between proxy and self-report as evidence that autistic people are incapable of accurate self-report, most autistic people are able to reflect and reliably report on their internal experiences (Kim and Lecavalier 2024). However, the measures themselves play a role, and it is important that researchers carefully consider whether the instruments they use are sufficiently validated to support their findings, including specifically with autistic people if originally developed for the general population.

The Patient-Reported Outcomes Measurement Information System (PROMIS) Family Relationships scale has demonstrated strong psychometric properties for general population adolescents (Bevans et al. 2017). Although the parent proxy version has been successfully used with parents of autistic children ages 5–12 (Schwartz et al. 2018), further psychometric validation of the self-report PROMIS Family Relationships scale with autistic adolescents is necessary. Specifically, researchers cannot presume that a measure which has been validated for the general population will function identically with autistic teenagers. Measurement invariance tests whether a scale measures an underlying construct the same way between groups, or more specifically, whether any differences in observed scores are explained by true group differences (Chen 2008). To that end, we evaluated measurement invariance of the PROMIS Family Relationships scale among autistic and general population teens.

## 2 | Methods

### 2.1 | Participants

The present study is a secondary data analysis of two datasets in which autistic and general population adolescents, ages 14–17, and their parents participated via an online survey. See Table 1 for participant demographics. All recruitment and study procedures were approved by the Institutional Review Board at the Children’s Hospital of Philadelphia (CHOP).

#### 2.1.1 | Autistic Sample

The autistic sample consisted of self-report data of 138 teens from Graham Holmes et al. (2020). Full recruitment details are reported in the original study; in short, parents and teens were recruited through CHOP and the Interactive Autism Network, and eligibility included parent-reported medical autism diagnosis and self-reported ability to complete the survey independently. The sample included approximately three times as many males (*n* = 98; 71.01%) as females (*n* = 37; 26.81%), with three teens identifying as transgender. The sample was predominantly white (*n* = 129; 93.48%) and non-Hispanic/Latino (*n* = 130; 94.20%).

#### 2.1.2 | General Population Sample

Our comparison group consisted of 403 teens from the PROMIS pediatric normative sample (Irwin et al. 2010). Parents and teens were recruited through the GfK Knowledge Panel; full recruitment details are reported in the original study. The sample was evenly split between males (*n* = 202; 50.12%) and females (*n* = 199; 49.38%); the general population sample was asked to identify as male or female without specifying cis- or transgender. As the panel aimed to be nationally representative, the gender ratio was balanced and racial/ethnic makeup more closely matched the broader US population (*n* = 322; 79.90% white and *n* = 343; 85.11% non-Hispanic/Latino).

### 2.2 | Measure

We used the short form pediatric self-report version of the PROMIS Family Relationships scale, which consists of eight items scored on a scale of 1 (Never) to 5 (Always), with higher scores indicating a more positive relationship with one’s family (Bevans et al. 2017). The PROMIS scales are unidimensional QoL measures available through the National Institutes of Health (NIH; Reeve et al. 2007) and were validated with representative samples of the US population (Cella et al. 2010). The scales can be administered individually or as part of a wider battery, such as the PAB-L (Graham Holmes et al. 2020).

### 2.3 | Data Analysis

We first fit confirmatory factor analysis (CFA) models to our samples using the *lavaan* package in R (Rosseel 2012). While a variety of models were considered, we ultimately proceeded with a unidimensional model, which was the best choice for alignment with theory, parsimony, and fit. We identified models by setting factor variance to 1. Given the ordinal nature of the PROMIS scales and low item-level missingness (15 items across 11 general population participants, none for autistic sample), we used the robust diagonally weighted least squares estimator with pairwise deletion for missing values. To avoid potentially inflated standard fit indices (Xia and Yang 2019), we used robust categorical maximum-likelihood estimators (Savalei 2021). Because RMSEA is often elevated for models with few degrees of freedom with few items and factors (Cheung and Rensvold 2002)—like the PROMIS scales, even for their validation samples (Bevans et al. 2017)—we anticipated higher RMSEA values and interpreted fit statistics holistically. To address inadequate baseline model fit, we considered model modification indices and correlated error terms where theoretically justified (Jorgensen 2017). For most items, no autistic teens endorsed the lowest response; for those items, the two lowest categories were collapsed for both groups (Tsai et al. 2024). To maximize our ability to detect non-invariance, we did not collapse categories for items where there were sufficient responses in each category.

Next, we fit a series of increasingly restrictive multigroup CFA models using *lavaan* (Rosseel 2012) and *semTools* (Kline 2015; van de Schoot et al. 2012). Configural invariance was assessed by allowing all factor loadings and item intercepts to vary freely, confirming that the overall factor structure of the measure fit well for both groups. Next, metric invariance was assessed by constraining factor loadings to be equivalent across groups while allowing item intercepts to vary freely. Metric invariance was established if there was no significant difference in fit between the metric and configural CFA models, indicating that there was no difference in the pattern of item loadings across groups. Finally, scalar invariance was assessed by constraining both factor loadings and item intercepts to be equivalent across groups. Scalar invariance was established if there was no significant difference in fit between the scalar and metric CFA models.

In lieu of rule-of-thumb cutoffs, which vary in the literature and may be affected by sample size, imbalanced group size, and level of invariance (Chen 2007; Cheung and Rensvold 2002; Hu and Bentler 1999; Meade et al. 2008), we used permutation testing (Jorgensen et al. 2018; Kite et al. 2018) to evaluate measurement invariance. By permuting group assignment and generating null distributions for each level of measurement invariance testing, we could empirically evaluate whether the Δχ^2^ and ΔCFI (D’Urso et al. 2021) between each model was significant. An a priori threshold of *α* = 0.01 was chosen for statistical significance to reduce false positives and due to the moderate power of our sample to detect sizable invariance at this significance level (Kite et al. 2018; Lakens et al. 2018). If *p*-values were non-significant (≥ 0.01), fit for the more restrictive model did not deteriorate, thus demonstrating measurement invariance.

## 3 | Results

### 3.1 | Internal Reliability

A summary of reliability and means by group is presented in Table 2. The PROMIS Family Relationships scale demonstrated excellent internal consistency across both the autistic (*ω*_T_ = 0.96) and general population (*ω*_T_ = 0.95) groups.

### 3.2 | Confirmatory Factor Analysis

Because the unmodified unidimensional Family Relationships model demonstrated borderline fit (results reported in Supporting Information), we examined modification indices for opportunities to improve fit. Since the items “My family treated me fairly” and “My family listened to me” capture highly related concepts that shared common variance, we correlated error terms for those items. Fit for the modified model was excellent for the combined sample (*χ*^2^ = 74.466, CFI = 0.996, TLI = 0.994, SRMR = 0.022). Fit was good for the autistic sample (*χ*^2^ = 52.629, CFI = 0.949, TLI = 0.925, SRMR = 0.044) and excellent for the general population sample (*χ*^2^ = 61.794, CFI = 0.980, TLI = 0.970, SRMR = 0.025). Detailed results are reported in Table 3.

### 3.3 | Measurement Invariance

Results of the measurement invariance analyses for the Family Relationships scale across groups are presented in Table 4. As shown by non-significant permutation testing (all *p*s > 0.01) at each level of increasing equality constraints, the Family Relationships scale demonstrated configural, metric, and scalar invariance.

## 4 | Discussion

Evaluating measurement invariance is an important step in the psychometric validation necessary to use instruments developed for the general population in clinical and research contexts with autistic people. Autistic people may interpret items or response options differently from their non-autistic peers, and in some cases, the construct of interest is intertwined with the autistic experience such that an instrument does not measure the same construct as in the general population (Nicolaidis et al. 2020). Particularly with a subjective outcome like quality of family relationships (Schiltz et al. 2024), before comparing findings across groups, it is vital to show that researchers are truly measuring the same construct in both groups and that the chosen measure functions similarly in a new population as the one in which it was initially tested. While the parent-proxy PROMIS Family Relationships scale has been validated for use with parents of autistic children (Schwartz et al. 2018), our study is the first to present evidence that the self-report version short form functions similarly for autistic teens and general population teens.

Self-report is vital to our understanding of autistic people’s lived experience and underutilized in autism research, often due to concerns that autistic people are less able to reflect and report on their own experiences than their non-autistic peers—an assumption rooted in ableism (Schiltz et al. 2024). Psychometrically sound PROMs facilitate research and clinical interventions which center the voices of autistic people themselves. The necessity of self-report holds especially true for teenagers, who are working to increase independence and define their own identity and goals. Particularly in the transition to adulthood, autistic adolescents note the influence of family on subjective and objective QoL (Lam et al. 2020; Øverland et al. 2024), and supportive family relationships constitute an important factor in later outcomes (Test et al. 2014). In this life stage, parent proxy report may not capture the depth of the individual’s opinion on their own life, so researchers require a well-validated PROM shown to work well with autistic teens.

Our results show configural, metric, and scalar invariance for the PROMIS Family Relationships scale. Because the scale demonstrated scalar invariance, we can conclude that the construct is being measured in a similar way for autistic and general population teens. Thus, researchers can confidently answer questions within the group of autistic teens or those that require comparing scores across autistic and non-autistic groups.

Future research with the PROMIS Family Relationships scale should encompass two paths: further psychometric validation and applied research. Since autistic people across the lifespan value family relationships as a QoL outcome (Graham Holmes et al. 2020), follow-up work should aim to establish measurement invariance for this scale with other age groups, equating scores in adolescents with those in younger children and adults. Furthermore, while self-report is ideal when assessing subjective experiences, there are many autistic people for whom parent proxy report may remain more realistic, such as those who are very young or unable to read or respond verbally to questions about their QoL. Thus, future research should aim to establish “equivalent measures” of various constructs that span multiple reporters in addition to investigating the validity of each alternative measure with subgroups of the autistic population to ensure psychometric adequacy.

Now that the PROMIS Family Relationships scale has been validated for autistic adolescent self-report, researchers have a useful tool to directly ask autistic teens about their lived experience to understand the impact of family relationships on autistic QoL. The existing body of literature on autism and family relationships has been historically one-sided, largely focused on parents and sometimes siblings. Reporting from only one half of a dyadic dynamic perpetuates an ableist construction of knowledge that privileges the neurotypical experience. While the pediatric parent proxy PROMIS Family Relationships scale has been used in autism research (Schwartz et al. 2018), to our knowledge, our study is the first to investigate self-report with autistic teens. Autistic teens deserve for their voices to be prioritized in research that claims to be about them. Applied research should seek input from autistic adolescents themselves to identify protective factors for family relationships or adapt family-related therapeutic interventions.

### 4.1 | Limitations

We note two measure-related limitations to our study. First, it should be acknowledged that although our results show the PROMIS Family Relationships scale measures the same underlying construct of family relationship quality in the same way among autistic teens as their general population peers, measurement invariance cannot reveal whether it captures the most important aspects of that construct for autistic people, and researchers should strive to incorporate autistic perspectives in future scale development. Additionally, collapsing the two lowest response categories to account for no “Never” responses in the autistic group limits our ability to evaluate the differential response threshold of that specific item category for those items between groups.

Our study also has some sample limitations related to the nature of secondary data analysis. First, we intentionally refer to the PROMIS validation sample as “general population” rather than “typically developing” or “non-autistic” because autism diagnoses were not reported for that sample. Given the representative nature of the sample drawn from the broader US population, we expect that a few autistic people may have been included, but true diagnostic status will remain unknown. Relatedly, we were unable to characterize participants in depth on cognitive functioning, verbal ability, or adaptive behavior skills. Future work which assesses these factors could further validate the generalizability of our findings accounting for the diversity in abilities among autistic people.

Our samples also share limitations commonly recognized as areas for growth across autism research as a whole, including racial and ethnic makeup more white and non-Hispanic/Latino than the current US population (Giwa Onaiwu 2020; Jones et al. 2020; West et al. 2016); an imbalanced sex and gender ratio, which reflects diagnostic patterns but does not fully encompass the increased gender diversity noted on the autism spectrum (Mittertreiner et al. 2024); and underrepresentation of minimally verbal autistic people and/or those with co-occurring intellectual disability (Maenner et al. 2021). While we did not exclude participants based on verbal ability or IQ, given the design of the questionnaire, we were able to establish measurement invariance only for teens in both groups capable of independent self-report. Moreover, the autistic group was not large enough to evaluate differential item functioning (DIF) along any of these axes of sample diversity. Although the autistic group differed significantly from the general population group on demographic factors, it is worth noting that previous evidence for this measure has demonstrated no DIF by ethnicity, race, or gender (Bevans et al. 2017). Future studies may benefit from oversampling underrepresented groups to more fully represent autistic perspectives, as well as performing formal DIF analyses across subgroups. In addition, researchers should investigate accommodations to make PROMIS questionnaires more accessible to individuals who might struggle with standard self-report.

## 5 | Conclusion

This study is the first to investigate measurement invariance of the pediatric self-report PROMIS Family Relationships scale with autistic and general population adolescents. By establishing scalar invariance, this study showed that this scale is valid for use with autistic teens and scores can be meaningfully compared across groups. With an accurate tool to investigate autistic teens’ family relationships, researchers should follow a call to action to prioritize and include autistic perspectives rather than solely relying on proxy reports.

## Supplementary Material

Supplementary Materials

Additional supporting information can be found online in the Supporting Information section. **Data S1:** Supporting Information.

## Figures and Tables

**TABLE 1 |:** Demographic information.

Variable	Autistic group (%)	General population group (%)	*p* ^a^

Totals	138	403	
Age (mean[SD])	15.48 (1.10)	15.50 (1.09)	0.85
14	34 (24.63%)	96 (23.82%)	
15	36 (26.09%)	104 (25.81%)	
16	36 (26.09%)	109 (27.04%)	
17	32 (23.19%)	94 (23.33%)	
Gender			
Female	37 (28.81%)	199 (49.38%)	< 0.01
Male	98 (71.01%)	202 (50.12%)	
Transgender	3 (2.17%)	N/A^b^	
Missing	0 (0.00%)	2 (0.50%)	
Race			
Asian	0 (0.00%)	15 (3.72%)	0.01^c^
Black	4 (2.90%)	29 (7.20%)	
Native American	0 (0.00%)	3 (0.74%)	
White	129 (93.48%)	322 (79.90%)	
Other or more than one race	5 (3.62%)	34 (8.44%)	
Ethnicity			
Hispanic or Latino/a	8 (5.80%)	60 (14.89%)	0.01
Not Hispanic or Latino/a	130 (94.20%)	343 (85.11%)	

a Two-sample *t*-test.

b Not reported for general population.

c Pearson’s chi-squared test.

**TABLE 2 |:** Domain summaries by group.

Group	*n*	*ω*_T_	M (SD)

Autistic teens	138	0.96	50.66 (9.71)
General population teens	403	0.95	47.92 (8.96)

Abbreviation: *ω*_T_, omega total (model-based reliability).

**TABLE 3 |:** Confirmatory factor analysis model results.

Group	*χ* ^2^	df	*p*	CFI	TLI	SRMR	RMSEA (90% CI)

Combined sample	74.466	19	< 0.001	0.996	0.994	0.022	0.098 (0.076–0.122)
Autistic teens	52.629	19	< 0.001	0.949	0.925	0.044	0.156 (0.097–0.215)
General population teens	61.794	19	< 0.001	0.980	0.970	0.025	0.093 (0.066–0.120)

**TABLE 4 |:** Model comparison statistics (w/permutation).

	Model comparison statistics				
	
Model	Comparison	Δ*χ*^2^	*p*	ΔCFI	*p*

1. Configural	Null vs. 1	53.777	0.064	> 0.999	0.063
2. Metric	1 vs. 2	23.257	0.186	< −0.001	0.184
3. Scalar	2 vs. 3	4.704	0.232	< 0.001	0.449

## Data Availability

The data that support the findings of this study are available on request from the corresponding author. The data are not publicly available due to privacy or ethical restrictions.

## Summary

Autistic people and their families value research about quality of life, including family relationships. When researchers use tools that were originally developed for the general population, rather than those designed specifically for autistic people, they must first make sure that the tool measures the same thing in the same way for autistic people. This study tested whether a tool that works well for the general population, the PROMIS Family Relationships scale, also works well for autistic teens. We found that the scale did measure the same thing in the same way for autistic teens, meaning that researchers can confidently do research using this scale with autistic teenagers and compare answers from both groups. This is important because most research on autism and family relationships asks questions of parents, but this scale can be used directly with autistic teens themselves to ask about their experiences.
