# Supplementary material for: Measurement Invariance of the PROMIS Family Relationships Scale Among Autistic and General Population Adolescents
Source: Autism Res. 2026 Jan 8;19(2):e70161. doi: 10.1002/aur.70161 (PMC12945458; doi:10.1002/aur.70161)
Supplement: Supplementary file 1 — Data S1: Supporting Information. [file AUR-19-0-s001.docx]

**Supplementary Materials**

| ***Table S1*. *Confirmatory Factor Analysis Model Results - Unmodified*** | | | | | | | |
| --- | --- | --- | --- | --- | --- | --- | --- |
| **Group** | ***X^2^*** | ***df*** | ***p*** | ***CFI*** | ***TLI*** | ***SRMR*** | ***RMSEA (90% CI)*** |
| Combined Sample | 89.706 | 20 | <.001 | .971 | 0.960 | .026 | .108 (.807-.130) |
| Autistic Teens | 75.703 | 20 | <.001 | .907 | 0.869 | .057 | .205 (.152-.261) |
| General Population Teens | 63.065 | 20 | <.001 | .978 | 0.970 | .025 | .093 (.068-.120) |
